# Towards clinical application of image mining: a systematic review on artificial intelligence and radiomics

**DOI:** 10.1007/s00259-019-04372-x

**Published:** 2019-06-18

**Authors:** Martina Sollini, Lidija Antunovic, Arturo Chiti, Margarita Kirienko

**Affiliations:** 1 Department of Biomedical Sciences, Humanitas University, Via Rita Levi Montalcini 4, 20090 Pieve Emanuele, Milan, Italy; 2 Nuclear Medicine, Humanitas Clinical and Research Center IRCCS, Rozzano, Milan, Italy

**Keywords:** Radiomics, Artificial intelligence, Texture analysis, Imaging, Systematic review, Trial phases

## Abstract

**Purpose:**

The aim of this systematic review was to analyse literature on artificial intelligence (AI) and radiomics, including all medical imaging modalities, for oncological and non-oncological applications, in order to assess how far the image mining research stands from routine medical application. To do this, we applied a trial phases classification inspired from the drug development process.

**Methods:**

Among the articles we considered for inclusion from PubMed were multimodality AI and radiomics investigations, with a validation analysis aimed at relevant clinical objectives. Quality assessment of selected papers was performed according to the QUADAS-2 criteria. We developed the phases classification criteria for image mining studies.

**Results:**

Overall 34,626 articles were retrieved, 300 were selected applying the inclusion/exclusion criteria, and 171 high-quality papers (QUADAS-2 ≥ 7) were identified and analysed. In 27/171 (16%), 141/171 (82%), and 3/171 (2%) studies the development of an AI-based algorithm, radiomics model, and a combined radiomics/AI approach, respectively, was described. A total of 26/27(96%) and 1/27 (4%) AI studies were classified as phase II and III, respectively. Consequently, 13/141 (9%), 10/141 (7%), 111/141 (79%), and 7/141 (5%) radiomics studies were classified as phase 0, I, II, and III, respectively. All three radiomics/AI studies were categorised as phase II trials.

**Conclusions:**

The results of the studies are promising but still not mature enough for image mining tools to be implemented in the clinical setting and be widely used. The transfer learning from the well-known drug development process, with some specific adaptations to the image mining discipline could represent the most effective way for radiomics and AI algorithms to become the standard of care tools.

**Electronic supplementary material:**

The online version of this article (10.1007/s00259-019-04372-x) contains supplementary material, which is available to authorized users.

## Introduction

The “Artificial Intelligence (AI) winter” [1] is over. AI and radiomics approaches applied to medical images for the non-invasive characterisation of diseases (i.e., image mining) have remarkably increased in recent years. The first reports on AI and radiomics applied to medical images date back to 1963 [2] and 1973 [3], respectively, but the enthusiasm of those years broke off quite soon. Recently, increasing amounts of electronic medical data, technological improvements, and health sustainability issues resulted in a renewed interest in both AI and radiomics applications.

Image mining is claimed to have a potentially huge clinical relevance with the possibility to non-invasively diagnose, characterise and predict the outcome in almost all medical conditions. However, despite the amount of published studies, some issues including significance, goodness, and strength of the reported results are still to be addressed. Particularly, it is not clear how far image mining is from clinical practice.

Therefore, the aim of this systematic review was to analyse literature on AI and radiomics, including all medical imaging modalities, for oncological and non-oncological applications, in order to assess how far the image mining research stands from routine medical application. To do this, we applied a trial phases classification inspired from the drug development process.

## Material and methods

This systematic review was performed according to the PRISMA statement [4]. The PRISMA checklist is provided in Supplemental material.

### Search, eligibility criteria and study selection

The endpoint of the analysis was to assess the potential of AI applied to medical images and radiomics (i.e., image mining) to be implemented in clinics. Our search algorithm within the PubMed/MEDLINE database consisted of the combination of the following terms: “Artificial intelligence[Mesh]” OR “Radiomic” OR “Radiomics” AND/OR “Radiography[Mesh]”, OR “Ultrasonography[Mesh]” OR “Tomography[Mesh]”, OR “Image interpretation, computed-assisted[Mesh]”, OR “Multimodal imaging[Mesh]”, OR “Tomography, emission-computed[Mesh]”, OR “Photography[Mesh]”. No start date limit was used, and the search was extended until September 5th, 2018.

According to the scope of the review, we considered AI (i.e., algorithms that take images as inputs) and radiomics investigations aimed at relevant objectives in clinical practice: biological characterisation, risk stratification, treatment response prediction, toxicity prediction, and prognostication of a certain disease. The imaging modalities we considered were ultrasound, radiography, mammography, endoscopy, skin pictures, ocular fundus pictures, computed tomography (CT), magnetic resonance imaging (MRI), scintigraphy (either planar images, SPECT, or SPECT/CT) and positron emission tomography (PET) or PET/CT. Subsequently, we applied the following exclusion criteria: (a) articles not in the English language; (b) studies not within the field of interest; (c) guidelines, review articles and meta-analysis, editorials or letters, comments, and conference proceedings; (d) “in vitro”, phantom or animal studies; (e) case reports or small case series (≤ 10 patients); (f) studies involving healthy subjects; (g) research articles focused on methodological aspects (algorithm and/or software development and/or comparison; evaluation/comparison of method(s) for parameters optimization, segmentation and features extraction; test–retest studies); (h) testing data (not medical images) as input for AI algorithm(s); (i) radiomics studies evaluating descriptors of shape and size or image intensity histogram only (i.e., not textural features); (j) lack of validation in a clinical setting; (k) lack of conventional metrics (i.e., sensitivity, specificity, accuracy, and/or hazard ratio, and/or recall, and/or AUC, and/or C-index) for the report of validation results.

Two reviewers (MK and MS) independently performed an initial screening of the identified titles and abstracts applying the inclusion/exclusion criteria. The discrepancies were resolved by a third reviewer (LA). The decision rule for consensus was simple majority. Then, the reviewers retrieved the full-text reports of the selected abstracts and, subsequently, performed an independent second-step selection.

### Quality assessment of the literature

Quality assessment of selected papers was performed according to the QUADAS-2 criteria, assessing 4 domains: (1) patient selection, (2) index test, (3) reference standard, and (4) flow and timing [5]. The signalling questions for each QUADAS-2 domain were tailored for the aim of this review as detailed in Table 1. This evaluation assigned the risk of bias to a study and ranked it as low (score = 2), high (score = 1), or indeterminate (score = 0) for each domain. We calculated the overall QUADAS-2 score as the sum of the scores. The appropriateness of statistical analysis was defined considering two aspects. First, the total number of patients analysed was considered appropriate if at least five patients/feature (after feature selection, if performed) were included in a radiomics study; while AI studies with more than 50 patients were considered as acceptable quality. The sample size criterion for radiomics studies was used adapting the conventional rule for multiple regression: the number of data points (i.e., observations or cases) should be considerably more than 5–10 times the number of variables [6]. At least ten patients per feature have been recommended in radiomics studies [7, 8]. The sample size criterion for AI studies was established assuming that at least 50 patients are needed to train and validate an algorithm, minimising the effects of overfitting and improving the quality of performance metrics, similarly to what is recommended for biomarker discovery [9]. Second, we assessed the balance in the number of patients between the subgroups (e.g. number of patients with benign vs malignant lesions in a study aimed at differential diagnosis); an imbalance of more than 2/3 was considered inappropriate.

**Table 1:** Description of the QUADAS-2 criteria used for the qualitative assessment

Patient selection	Index test	Reference standard	Flow and timing
Signalling question 1: Was the statistical management adequate?	Signalling question 1: Were the imaging acquisition protocol and the segmentation method(s) detailed?	Signalling question 1: Was the reference standard adequate?	Signalling question 1: Was there an appropriate interval between index test and reference standard?
Signalling question 2: Were the inclusion/exclusion criteria specified?	Signalling question 2: Was the image processing approach detailed?		
Signalling question 3: Was the type of study (retrospective or prospective) specified?	Signalling question 3: Was the validation independent (i.e., no internal)?		

### Phases classification criteria

**Fig. 1:**
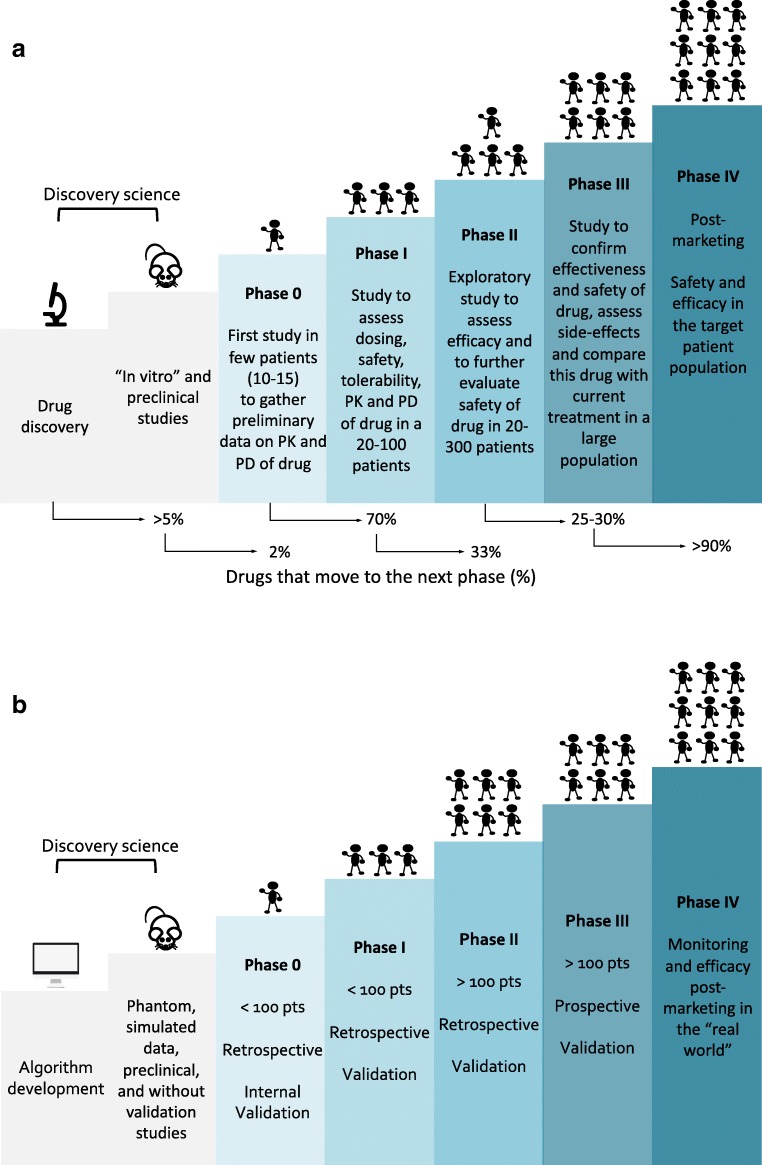
Trial phases. Trials classification for the drug development process (**a**) and for the proposed image mining tools development process (**b**). *PK* pharmacokinetics, *PD* pharmacodynamics

We developed the phases classification criteria for image mining studies, inspired by the classification applied to the clinical trials (Fig. 1). The parameters for phase categorisation included: sample size, type of study (retrospective/prospective), type of validation approach (internal/independent), and the development stage (pre-/post-marketing). Figure 1 reports the classification criteria in detail. We assigned each selected article to a phase: from 0 to IV.

### Statistical analysis

A database was created for the qualitative synthesis of the papers and the studies’ results. We recorded the results obtained in the validation set only. In case of multiple aims within the same article, the primary one was considered. If image mining was applied to different imaging modalities within the same article (e.g., PET and CT), results were recorded for each one. If different approaches were tested within the same paper (e.g., models derived from random forest and support vector machine), the best in terms of diagnostic performance was analysed. The articles reporting identical or very similar sample size, research hypothesis, methodology and results sharing with almost the same authorship—“salami” publishing [10]—were excluded to avoid overlap in the study population and results redundancy. Accordingly, in case of a series of articles considered as “salami”, the one with the larger sample size was evaluated and the other(s) excluded. Papers with a QUADAS-2 ≥ 7 were included in the quantitative analysis. Descriptive statistical measures were used to summarise the data. Excel ® 2017 (Microsoft®, Redmond, WA) was used for analysis.

## Results

### Search, eligibility criteria and study selection

**Fig. 2:**
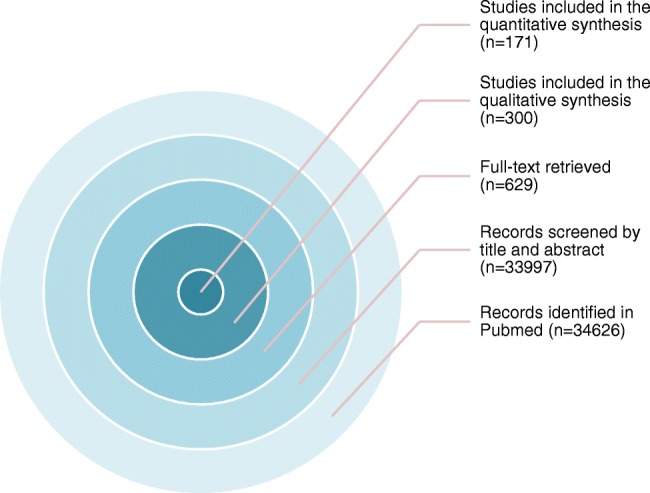
Study selection workflow

**Fig. 3:**
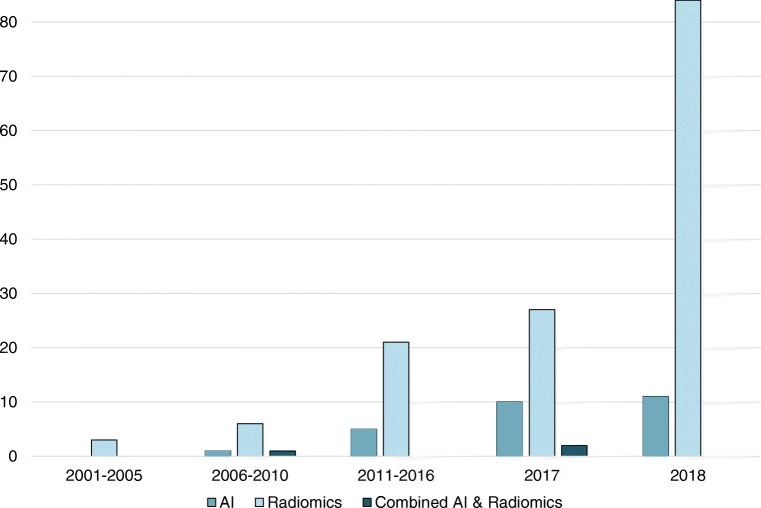
Trend of the published studies on artificial intelligence (AI), radiomics and the combined approaches radiomics/AI

**Fig. 4:**
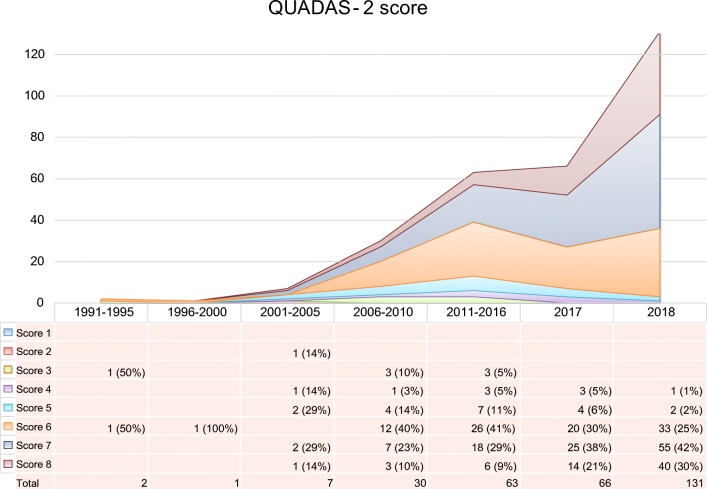
Trend of literature on image mining according to QUADAS-2 score, considering 300 selected studies

**Fig. 5:**
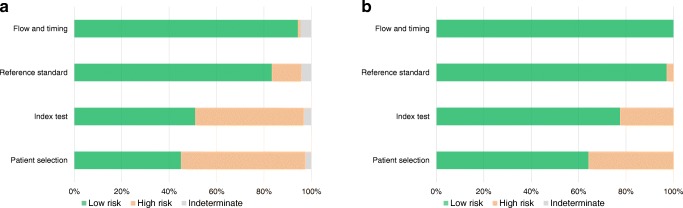
QUADAS-2 assessment results. Distribution of the articles tabulated by the four QUADAS-2 domains for the 300 studies selected applying the inclusion/exclusion criteria (**a**) and for the 171 studies scored ≥7 (**b**)

**Fig. 6:**
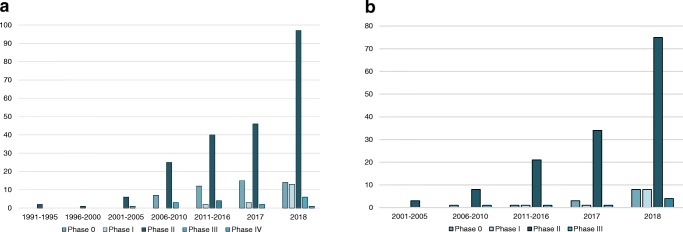
Trend of literature on image mining according to trial phases classification, considering 300 selected studies (**a**) and the 171 high-quality studies (**b**)

**Fig. 7:**
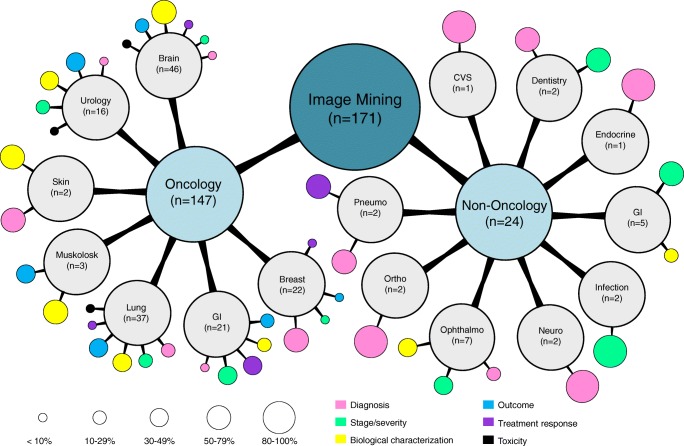
Radiomics and artificial intelligence literature summary by disease and clinical setting

**Fig. 8:**
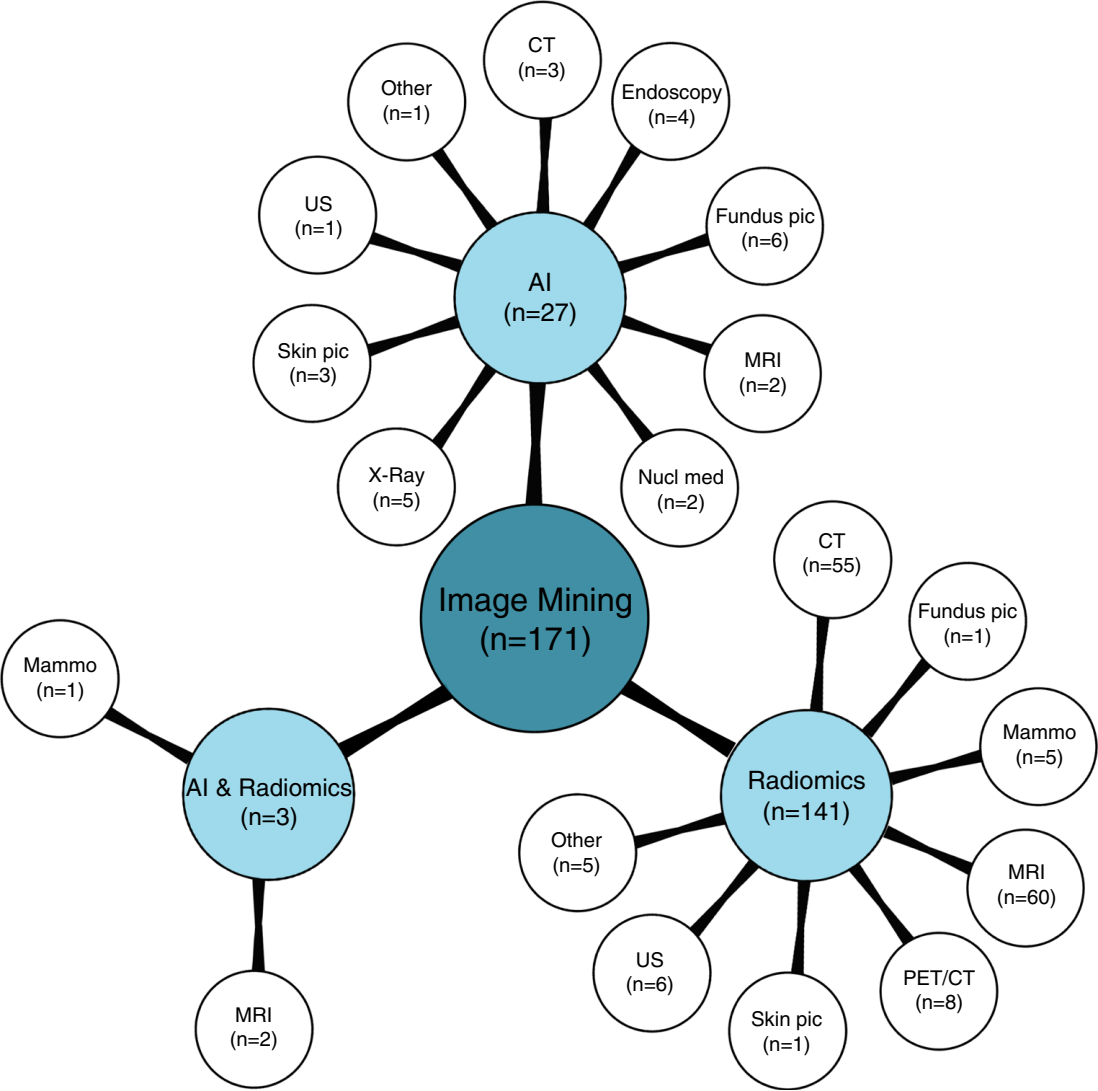
Radiomics and artificial intelligence literature summary by image mining approach and imaging modality

**Fig. 9:**
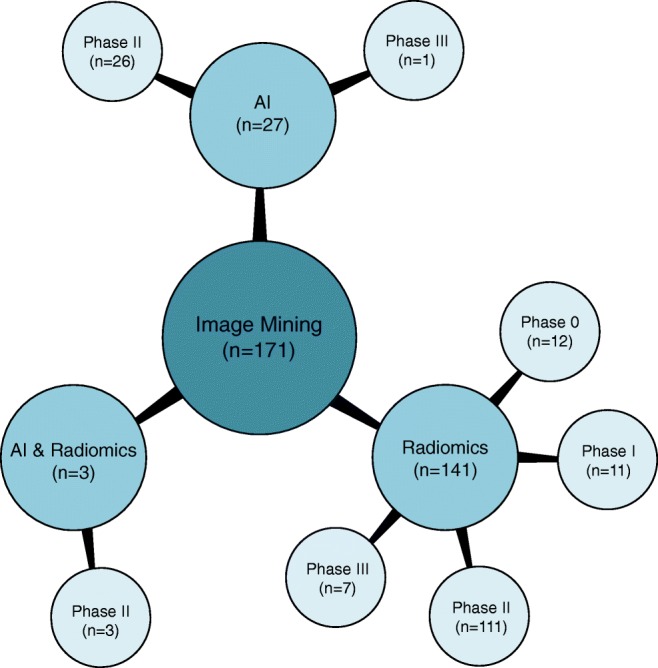
Radiomics and artificial intelligence literature summary by image mining approach and phase classification

Overall 34,626 articles were retrieved using the search algorithm. Subsequently, 33,997 papers were excluded reviewing titles and abstracts and applying the inclusion/exclusion criteria. Five papers, suspected to be a “salami” publishing, were excluded. Figure 2 summarises the research process. Table S1 reports a qualitative summary of the selected 300, including the 171 high quality articles. In recent years, a striking increase in the number of papers published on image mining occurred. In fact, especially in 2017 and 2018, 66 (22%) and 131 (44%) articles, respectively, were published. Figure 3 shows the literature trend. The vast majority (more than 80%) come from the oncology field. However, more than 50% of the studies included in the qualitative analysis have been assigned a high risk of bias (Fig. 4). Accordingly, a substantial proportion 129/300 (43%) studies have been scored as having a considerable risk of bias, mainly in the “index test” and “patient selection” domains (QUADAS-2 ≤ 6) (Fig. 5). Table S2 reports a qualitative summary of the 171 high-quality (QUADAS ≥7) papers. The temporal trend of the literature according to the phase of the study is shown in Fig. 6. Figures 7, 8, and 9 represent the graphical syntheses of the high-quality articles (QUADAS-2 ≥ 7) considered from three different points of view: the clinician, the imager, and the researcher. Quantitative synthesis is summarised in Table 2. The main results of the phase III studies are reported in Table 3.

**Table 2:** Quantitative synthesis of the 171 selected articles

Approach	Domain	Disease	Outcome	Imaging modality	Images, *n*	Type of validation	Phase	Reference
AI	Neurology	Alzheimer	Diagnosis	MRI	834	Internal	II	[11]
		Parkinson		Scintigraphy	175			[12]
	Cardiovascular	CAD		Scintigraphy	308	Split-sample	II	[13]
	Dentistry	Caries		Radiography	3000		II	[14]
		Teeth			1740			[15]
	Endocrinology	Acromegaly		Photo	1365		II	[16]
	GI	Liver	Stage/severity	US,CT	894	Geographical (*n* = 1) Split-sample (*n* = 1)	II, III	[17, 18]
		Polyp	Biological characterization	Endoscopy	1473	Split-sample (*n* = 1) Temporal (*n* = 1)	II	[19, 20]
	Infection	*H. pylori*	Diagnosis	Endoscopy	43,689	Split-sample	II	[21]
		Mycosis		Photo	50,925	Geographical		[22]
	Oncology	Lung	Diagnosis	CT	62,492	Split-sample	II	[23]
		Bone	Biological characterization	Radiography	500			[24]
		Brain		MRI	477			[25]
		Cervix		Colposcopy	485			[26]
		Skin		Skin pictures	129,450			[27]
		Esophagus	Treatment response	PET	107	Internal	II	[28]
	Ophthalmology	DR	Diagnosis	Fundus pictures	76,885	Geographical	II	[29]
			Biological characterization		430	Split-sample		[30]
			Stage/severity		92,961	Internal (*n* = 1) Split-sample (*n* = 1)		[31, 32]
		Macular disease	Biological characterization		109,312	Split-sample		[33]
			Stage/severity		133,821	Internal		[34]
	Orthopedics	Fracture	Diagnosis	Radiography	258,349	Internal (*n* = 1) Split-sample (*n* = 1)	II	[35, 36]
	Pneumology	COPD	Outcome	CT	10,655	Internal	II	[37]
Radiomics	GI	Liver	Stage/severity	US	144	Split-sample	III	[38]
	Oncology	Bladder	Biological characterization	MRI	61	Internal	0	[39]
			Stage/severity	CT, MRI	221	Temporal	II	[40, 41]
		Brain	Diagnosis	MRI	215	Internal (*n* = 1) Split-sample (*n* = 1)	0, I	[42, 43]
			Biological characterization		3732	Geographical (*n* = 1) Internal (*n* = 4) Split-sample (*n* = 11) Temporal (*n* = 4)	0 (*n* = 2), I (*n* = 1), II (*n* = 17)	[44–63]
			Stage/severity		286	Split-sample	II	[64]
			Treatment response		172			[65]
			Outcome		812	Geographical (*n* = 1) Internal (*n* = 3) Split-sample (*n* = 2) Temporal (*n* = 1)	0 (*n* = 2), I (*n* = 1), II (*n* = 4)	[66–72]
		Breast	Diagnosis	Mammography (*n* = 5), MRI (*n* = 4), US (*n* = 3)	2922	Geographical (*n* = 1) Internal (*n* = 9) Split-sample (*n* = 1) Temporal (*n* = 1)	0 (*n* = 1), II (*n* = 8), III (*n* = 3)	[73–84]
			Biological characterization	MRI	786	Internal (*n* = 2) Split-sample (*n* = 1)	I (*n* = 1), II (*n* = 2)	[85–87]
			Stage/severity		309	Split-sample	II	[88, 89]
			Treatment response		220	Internal, Split-sample		[90, 91]
			Outcome	MRI, mixed	407			[92, 93]
		Uterus	Biological characterization	MRI	160	Internal	III	[94]
			Stage/severity	PET	115	Split-sample	II	[95]
			Outcome	PET (*n* = 2), mixed (*n* = 2)	408		I (*n* = 2), II (*n* = 2)	[96–99]
		Colorectal	Biological characterization	CT	443	Split-sample	II	[100, 101]
			Stage/severity		1791	Temporal		[102–104]
			Treatment response	CT (*n* = 1), MRI (*n* = 3)	701	Geographical (*n* = 1), Internal (*n* = 1), Split-sample (*n* = 2)	0 (*n* = 1), II (*n* = 3)	[105–108]
			Outcome	MRI	108	Split-sample	II	[109]
		Esophagus	Stage/severity	CT (*n* = 2), MRI (*n* = 1)	608	Split-sample (*n* = 2) Temporal (*n* = 1)	II	[110–112]
			Treatment response	CT, MRI, PET	195	Internal (*n* = 2), Split-sample (*n* = 1)	0, I, II	[113–115]
			Outcome	CT	239	Split-sample	II	[116]
		GIST	Biological characterization		222		II	[117]
		Kidney			53	Internal	0	[118]
		Liver	Stage/severity		304	Split-sample	II	[119]
		H&N	Diagnosis	US	210	Internal	II	[120]
			Biological characterization	CT	969	Split-sample (*n* = 1) Temporal (*n* = 2)	I (*n* = 1), II (*n* = 2)	[121–123]
			Treatment response	MRI	120	Internal	II	[124]
			Outcome	CT (*n* = 2), MRI (*n* = 2), PET (*n* = 1)	1232	Geographical (*n* = 1) Split-sample (*n* = 3) Temporal (*n* = 1)	II (*n* = 4), III (*n* = 1)	[125–129]
			Toxicity	MRI	93	Geographical	I	[130]
		Mixed tumors	Biological characterization	CT	272	Split-sample	II	[131]
			Toxicity		32	Internal	0	[132]
		Lung	Diagnosis		1692	Internal (*n* = 2) Geographical (*n* = 1) Split-sample (*n* = 2)	II	[133–137]
			Biological characterization	CT (*n* = 13), PET (*n* = 2)	5235	Internal (*n* = 3) Geographical (*n* = 4) Split-sample (*n* = 4) Temporal (*n* = 3)	I (*n* = 1), II (*n* = 13)	[138–151]
			Stage/severity	CT	855	Internal (*n* = 2) Split-sample (*n* = 2)	II	[152–155]
			Treatment response		85	Internal	0	[156]
			Outcome	CT (*n* = 7), PET (*n* = 2), mixed (*n* = 2)	3125	Internal (*n* = 3) Geographical (*n* = 2) Split-sample (*n* = 5) Temporal (*n* = 1)	0 (*n* = 1), II (*n* = 9), III (*n* = 1)	[157–167]
			Toxicity	CT	192	Internal	II	[168]
		Ocular	Diagnosis	MRI	157	Split-sample		[169]
		Ovary		US	264	Geographical	III	[170]
		Pancreas		CT	103	Internal	II	[171]
		Prostate	Biological characterization	MRI	316	Split-sample		[172]
			Outcome		120	Geographical		[173]
		Sarcoma	Biological characterization		19	Split-sample	I	[174]
			Outcome	CT	150	Temporal	II	[175]
		Skin	Diagnosis	Skin pictures	162	Geographical		[176]
	Ophthalmology	Macular disease	Biological characterization	Fundus pictures	457	Temporal	II	[177]
	Pneumology	COPD	Diagnosis	CT	162	Split-sample	III	[178]
Combined AI and radiomics	Oncology	Breast	Diagnosis	Mammography	600	Split-sample	II	[179]
		Brain	Biological characterization	MRI	119	Internal	II	[180]
			Outcome		112	Geographical	II	[181]

*AI* artificial intelligence, *AUC* area under the curve, *CAD* coronary artery disease, *COPD* chronic obstructive pulmonary disease, *CT* computed tomography, *H&N* head and neck, *GI* gastrointestinal, *GIST* gastrointestinal stromal tumors, *MRI* magnetic resonance imaging, *PET* positron emission tomography, *US* ultrasonography

**Table 3:** Summary of the results of the phase III trials on image mining (*n* = 8)

Approach	Domain/disease	Outcome	Imaging modality	Images, *n*	Main results	Reference
AI	GI/Liver	Stage/severity	US	398	AUC = 0.85	[17]
Radiomics				144		[38]
	Oncology/Breast	Diagnosis	US	147	AUC = 0.93	[80]
	Oncology/Cervix	Biological characterization	MRI	160	Accuracy = 69%	[94]
	Oncology/H&N	Outcome	CT	172	C-Index = 0.73	[127]
			PET		C-Index = 0.71	
	Oncology/Lung		PET	312	C-Index = 0.59	[163]
	Oncology/Ovary	Diagnosis	US	264	Sensitivity = 98%	[170]
					Specificity = 88%	
	Pulmonary/COPD		CT	162	AUC = 0.89	[178]

*AI* artificial intelligence, *AUC* area under the curve, *COPD* chronic obstructive pulmonary disease, *CT* computed tomography, *H&N* head and neck, *GI* gastrointestinal, *MRI* magnetic resonance imaging, *PET* positron emission tomography, *US* ultrasonography

## Discussion

The present systematic review is the first assessing the potential for implementation of image mining tools in clinical practice, by means of classification of the literature in development phases. Despite the amount of literature on image mining with a validation analysis, more than 90% of studies were classified as phase 0, I or II (i.e., retrospective). Collectively, their results were uncertain in terms of significance, goodness, and strength and their generalisability weak. Even among the studies with a QUADAS-2 ≥ 7, only 4.6% were categorised as phase III studies. As it emerges from the present systematic review, the results are promising but still not mature enough for clinical implementation and widespread use of image mining tools. Nonetheless, the study quality has increased in recent years.

Because of the paucity of phase III and IV studies, we did not proceed to a meta-analysis. Therefore, no definitive conclusion can be drawn on which approach among radiomics and AI should be preferred. AI techniques, in particular convolutional neural networks, have the advantage over radiomics of not requiring tumour segmentation, feature calculation and selection. These steps are even more critical in tiny lesions that have to be submitted to radiomics processing. On the other hand, vast cohorts are crucial for a robust AI-based model development that require big efforts to be collected and analysed. Also, an unbiased reference standard, not always easy to obtain, should be chosen to ensure AI model reliability. The combined radiomics/AI strategy is at its early stages [179–183] and the complementary role of radiomics and AI techniques should be addressed [184]. Which is the best image mining approach is still an open question.

Similar to the other “omics” domains (e.g., proteomics), few of the image mining biomarkers reached clinical practice [9]. The translation of image mining research in the clinical arena is limited by the huge variability of the methods used for image analysis, together with the impasse to reproduce the results when tested in a different cohort of patients. Validation is a critical issue. Theoretically, the validation analysis of a successful model should provide consistent performance measures to those obtained in the training process. Thereafter, results obtained in the validation cohort should be confirmed by the test-independent validation. Finally, the proposed approach should be effective for the indication within the “real world” population of patients the model has been developed for. The validation process may be internal (e.g., cross-validation and bootstrapping) or external (using data not used for training). Typically, the internal one, used for a preliminary evaluation or for the fine-tuning of the model under development, overestimate the performance [185]. In fact, the same cohort is used twice, once to choose the filtered subset and again to build a classification model resulting in the overfitting of the algorithm to the data [9]. The external validation may be performed using three different strategies: (i) temporal (i.e., data obtained in newly recruited patients), (ii) geographic (i.e., data collected in a different institution), and (iii) split-sample (i.e., data split from the entire dataset and kept untouched for the test). External validation is crucial to verify the generalisability of the results [185]; and the random patient selection is an essential prerequisite, as well as the balance in patient characteristics. Temporal or geographic validations should be preferred to the split-sample one. Particularly, the geographic validation, which accounts for technical variability aspects (scanners, acquisition parameters and protocols) [185], is expected to be more representative of the clinical setting.

We excluded a priori from the present analysis studies testing shape and size as well as histogram-based features since our aim was to assess the “maturity” of advanced image analysis at its’ full potential, entailing textural indexes derived from up to second-order features. Image analysis based on the gray level histogram only does not provide any information about the relative position of pixels/voxels to each other within the region of interest. Therefore, these features are not able to describe whether any low/high gray levels are positioned together, or if they are distributed between high/low-value gray levels [186].

In order to develop a valid and trustworthy image mining tool the cohorts in study (training, validation and test) should be representative of the target population. This means that the sample size should be big enough to minimise the effects of overfitting, be comprehensive of the “outliers”, and, consequently, be reliable when used for the assessment of unseen patients. We proposed that at least 50 patients should be included in AI studies, as also suggested by simulated analyses [9, 187, 188]. However, especially for deep learning approaches and complex tasks, much larger populations are needed. The effect of the sample size on the model performance has been already demonstrated. When a limited dataset (1000 samples) vs the complete dataset (>100,000) was used for retinopathy classifier development, the weighted error resulted as 12.7% vs 6.6%, respectively [33]. Moreover, the sample size was not the only criterion used to score papers. Accordingly, even if the number of patients was relatively low, only well-designed studies have been scored as having low risk of bias and included in the quantitative analysis.

In the present work we arbitrarily chose 100 samples as the threshold for trial phases categorisation (II vs III ad above). This was a conservative decision. The conventional rule of the necessity to include at least ten patients per tested variable if applied for phase classification would have led to downgrading of most of the studies. Considering the fact that generally a feature reduction strategy was put in place we chose 100 as a reasonable cut-off. When planning an image mining study, both statistical recommendations (sample size and prospective design) and clinical conditions’ variability should be considered in order to develop an algorithm on a dataset that realistically represents the target patient population. The sample size calculation has been estimated in only 3 out of the 171 papers [44, 88, 125]. The use of multiple images taken from the same patient should be limited since they are prone to be similar (or almost identical) with a negative impact on the generalisability potential. In fact, even if this process increases the sample size, the multiplied number of observations (or cases) not representative of the inter-patient variability, overestimates the model performance. Accordingly, data augmentation should be properly used to avoid overfitting, keeping in mind that it cannot completely overcome the requirement of a proper sample size.

To assess the appropriateness of the statistical analysis, we considered not only the sample size but also the number of patients within the subgroups (an imbalance of more than 2/3 was considered inappropriate). Imbalanced cohorts in image mining studies may lead to constitutively biased results, which confer higher uncertainty and poor generalisability [189]. Consequently, in the developmental phase, proper study design and analysis strategy using stratification, matching, weighting, covariate adjustment, or regression should be adopted. Therefore, imbalance per se does not prevent the use of radiomic and AI-based approaches when the prevalence of a disease or an outcome is very low [190].

The applicability of the image mining framework to rare diseases is still an issue because of the limited data availability for model development. Transversal platforms for sharing and analysis of images and data, as envisaged by some research groups [191], could represent a valuable strategy for the investigations in this field.

Presently, it is unrealistic to justify a medical decision by the output provided by a neural network or a radiomics feature/signature. Little or nothing is still known on the biological significance of the image-derived parameters. Correlations with tumour grading [39, 45, 46, 94, 100, 117, 118, 138, 174], inflammatory infiltrate [131], gene expression, mutation and molecular pathways [25, 44, 47–58, 85–87, 101, 121, 122, 139–142, 180] have been reported. Nonetheless, more should be learned about the functioning of AI and radiomics approaches in order to solve the “black box” problem and to understand the underlying clinical and/or molecular connotation. Imagers should be able to assess the reliability of image mining approaches and to manage independently the patient (i.e., the pilot, plane and passengers during a flight). This innovative attitude, which implies the acquisition of technical and informatics skills, will contribute to remove the “black box” uncertainties, and to promote image mining towards clinical practice.

Additionally, some technical barriers should be faced when considering implementing image mining tools into the every-day practice. These include a time-consuming workflow; uncertain reproducibility of results among different scanners, acquisition protocols, and image-processing approaches; regulatory issues concerning privacy and ethics; and data protection. Common efforts should be realised to accelerate the research path on these aspects, and to implement the technological infrastructure and make the tools easy to use. Privacy and ethics regulations may restrict data and image sharing for the purpose of research and every-day clinical practice. A shared strategy needs to be built up for the management of these aspects. These challenges are an opportunity to develop a reliable methodology able to provide controlled data collection and secure infrastructure, instead of gathering uncertain-quality datasets.

The need to provide reliable results has generated multiple initiatives and recommendations to achieve methodology standardisation and reproducibility [7, 185, 191–193]. The increasing awareness among researchers of the urgency to increase the quality of the investigations determined an increase in the number of phase III trials in the last 2 years. In the era of evidence-based medicine, rigorous research with strict rules is the only way forward to achieve clinical acceptance and become part of the “standard of care”. The research process should aim to address a clinical need through an adequate statistical strategy, prospective and multi centre studies, robust reference standards, and adequate timing. Independent validation is mandatory together with the clarification of the impact of the technical aspects on image mining models. These items imply that reproducible, strong, and, hopefully, excellent results will be achieved through an adequate research process. In this respect, a closer collaboration should be established among clinical researchers, algorithm developers and data scientists.

We foresee the transfer learning from the well-known drug development process, with some specific adaptations, to the image mining discipline as the most effective way for radiomics and AI algorithms to get into routine clinical practice and avoid a new glacial era of image mining in the next decades.

## Electronic supplementary material

ESM 1 (DOCX 31 kb)
